# The prevalence of fibromyalgia in axial spondyloarthritis

**DOI:** 10.1007/s00296-020-04621-5

**Published:** 2020-06-15

**Authors:** Gareth T. Jones, Bhadra Mallawaarachchi, Joanna Shim, Jonathan Lock, Gary J. Macfarlane

**Affiliations:** 1 Epidemiology Group, Aberdeen Centre for Arthritis and Musculoskeletal Health, University of Aberdeen, Aberdeen, UK; 2 Ministry of Health, Colombo, Sri Lanka

**Keywords:** Axial spondyloarthritis, Fibromyalgia, Prevalence, Meta-analysis

## Abstract

Comorbid fibromyalgia, in axial spondyloarthritis (axSpA) has been shown to influence disease activity and function, and quality of life. Although several papers exist, there is no comprehensive and robust systematic review to determine the prevalence of fibromyalgia in this patient group. Thus, the aim of the current study was to provide a definitive estimate of prevalence of fibromyalgia in axSpA, and in axSpA sub-classifications. A systematic literature search was conducted in Ovid MEDLINE, EMBASE, Evidence Based Medicine (EBM), and Cochrane Library, updated to April 2020, combining keywords and relevant MeSH headings, to identify papers reporting the prevalence of fibromyalgia in axSpA, or data from which this could be computed. This was then combined in a meta-analysis with data from the Scotland Registry for Ankylosing Spondylitis (SIRAS), a national axSpA register in Scotland. Data was pooled using random or fixed effects models where heterogeneity was greater or lesser than 75%. From 3401 manuscripts initially identified, 15 papers were included in the final review, plus SIRAS, giving data from 16 separate sources. The prevalence of fibromyalgia, among a total of 5214 patients, was 16.4% (95% CI 12.3–20.5%). Prevalence varied with axSpA sub-classification: ankylosing spondylitis: 13.8% (9.1–18.6%); MRI positive non-radiographic axSpA 20.3% (6.5–34.1%); and ‘clinical’ disease: 11.1% (6.0–16.2%). Overall, around 1 in 6 patients with axSpA also meet criteria for fibromyalgia. While estimates from individual studies vary, comorbid fibromyalgia represents a considerable burden across all sub-classifications of axSpA. This emphasises that focusing management solely on inflammatory disease in this patient group is unlikely to yield optimal improvements in quality of life.

## Introduction

Comorbid fibromyalgia, in persons with axial spondyloarthritis (axSpA), is of considerable and controversial interest. Several years ago, the US Food and Drug Administration expressed concern that patients with commonly occurring pain conditions, such as fibromyalgia, may be incorrectly diagnosed with non-radiographic axSpA (nr-axSpA), which in turn may lead to inappropriate treatment with biologic medications. We have previously shown that, among patients with axSpA, patients meeting the research criteria for fibromyalgia [1] report higher disease activity, poorer function and quality of life, and were more likely to report high fatigue and moderate or severe levels of mood disorder [2]. However, the likelihood of achieving treatment response among patients treated with TNF inhibition, did not differ between those who did and did not meet criteria for fibromyalgia [3].

In a recent systematic review of inflammatory arthritis more generally, Duffield and Miller et al. [4] identified nine studies reporting the prevalence of fibromyalgia in axSpA and/or ankylosing spondylitis (AS). However, their restricted search was unlikely to have identified all relevant publications. Indeed, while they report that nine articles studying axSpA were included, a preliminary search has revealed several additional important publications that contribute to the evidence base.

Using the maximum available data will decrease the uncertainty around the prevalence estimate. It also will provide the rheumatologist with the best evidence in terms of likely burden of fibromyalgia in their patient population. This may also help to direct resources in terms of patient management. Thus, the aim of the current study was to provide a definitive estimate of prevalence of fibromyalgia in axSpA through a comprehensive and systematic review of the literature, plus the addition of some new primary data.

Over the last decade, the Assessment of Spondyloarthritis International Society (ASAS) has led the way in challenging prior thinking about spondyloarthritis. Now recognised as a single disease entity, patients with axSpA can be classified into different groups: those with only clinical signs and symptoms, and no imaging-based evidence of sacroiliitis (ASAS clinical criteria), versus those with various clinical characteristics with imaging evidence of sacroiliitis (ASAS imaging criteria) [5]. The latter group is then further sub-classified into those with radiographic changes in the sacroiliac joints (AS) versus those with MRI evidence of sacroiliitis, but no x-ray changes. Thus, the second aim of the current study was to provide estimates of the prevalence of fibromyalgia stratified by different axSpA classification criteria.

## Methods

### Systematic literature review

The conduct and reporting of this meta-analysis were guided by the Preferred Reporting Items for Systematic Reviews and Meta-Analyses (PRISMA) statement [6]. Full-text peer-reviewed articles were eligible for inclusion based on the following criteria:

- Population: Persons with axSpA or AS—classified using explicit criteria, or clinical diagnosis. Studies presenting data on multiple patient groups were eligible, providing the data on axSpA and/or AS could be separately identified.
- Study design: Any study design. If longitudinal was available, data was taken from the timepoint with the greatest sample size, with respect to fibromyalgia data.
- Outcome: Either (a) Information on point or period prevalence of fibromyalgia—classified using explicit criteria, or clinical diagnosis; or (b) Data from which this could be computed. Studies presenting data on multiple outcomes were eligible, providing the data on fibromyalgia could be separately identified.

Studies were identified by searching electronic databases; Ovid MEDLINE, EMBASE, Evidence Based Medicine (EBM), and Cochrane Library up to December 2017. The search included the terms:

1. ankylosing spondylitis (mt) OR ankylosing spondylitis (tw) OR spondyloarthritis (mt) OR spondyloarthritis (tw) OR spondylarthritis (mt) OR spondylarthritis (tw) OR spondyloarthropathies (mt) OR spondyloarthropathies (tw) OR spondyloarthritides (mt) OR spondyloarthritides (tw) OR spondylitis (mt) OR spondylitis (tw)
2. fibromyalgia (mt) OR fibromyalgia syndrome (mt)
3. Boolean combination (1) AND (2)

Reference lists of included articles were also screened for inclusion, and update searches were then performed in June 2019 and April 2020. All the titles were initially screened by one reviewer, with 25% of titles checked by a second. This resulted in a single additional article taken forward to abstract review. Selected abstracts were then screened by one reviewer to identify manuscripts to be taken forward to full-text review. All excluded abstracts were checked by the second reviewer and no additional articles were taken forward to full-text review.

Full text articles of all selected abstracts were then screened, and a random sample of articles excluded at full text screening and all included full texts were independently reviewed by the second reviewer. One reviewer extracted relevant data from included studies which was independently cross-checked by a second reviewer for any transcription or interpretation errors. Any disagreements were resolved by discussion and consensus.

### Scotland Registry for Ankylosing Spondylitis (SIRAS)

The SIRAS protocol has been previously published [7]. In brief, patients with a clinical diagnosis of AS were recruited from rheumatology departments across Scotland. Eligible patients were those who had received a diagnosis of AS according to the modified New York criteria [8] or had been given a clinical diagnosis of AS by a consultant rheumatologist. Clinical data were collected from medical records, and participants completed postal questionnaires containing patient-reported measures. At the third follow-up (approximately 4 years after baseline) questions were included to be able to determine whether participants met the American College of Rheumatology (ACR) 2011 modification of the preliminary diagnostic criteria for fibromyalgia (also known as the modified 2010 criteria) [1]. The study was approved by the East of Scotland Research Ethics Service (reference: 16/ES/0030).

### Meta-analysis

For each paper, data on the reported prevalence and the sample denominator were used to compute the number of individuals with fibromyalgia. (Raw data was taken from the paper, if this was available.) Then, an exact 95% confidence interval around the prevalence estimate was computed using the method described by Clopper and Pearson [9]. The same approach was used for the SIRAS population.

Heterogeneity was quantified using the *I*^2^ statistic—i.e. the proportion of variation in prevalence estimates between studies attributable to heterogeneity, rather than chance. Where heterogeneity was judged to be high (*I*^2^ ≥ 75%) [10] a pooled estimate of fibromyalgia prevalence was obtained using a random-effects model, with inverse variance weighting. Where heterogeneity was moderate or low (*I*^2^ < 75%) a fixed effects model was employed. Separate models were produced based on different classifications of fibromyalgia: ACR-1990 criteria [16], ACR-2010 [17], ACR-2011 [1] and the Fibromyalgia Rapid Screening Tool (FiRST) [12]; and also for different classification criteria of axSpA: AS, as per the New York or modified New York criteria [8], or the ASAS imaging/clinical criteria [5].

## Results

### Systematic literature review

**Fig. 1:**
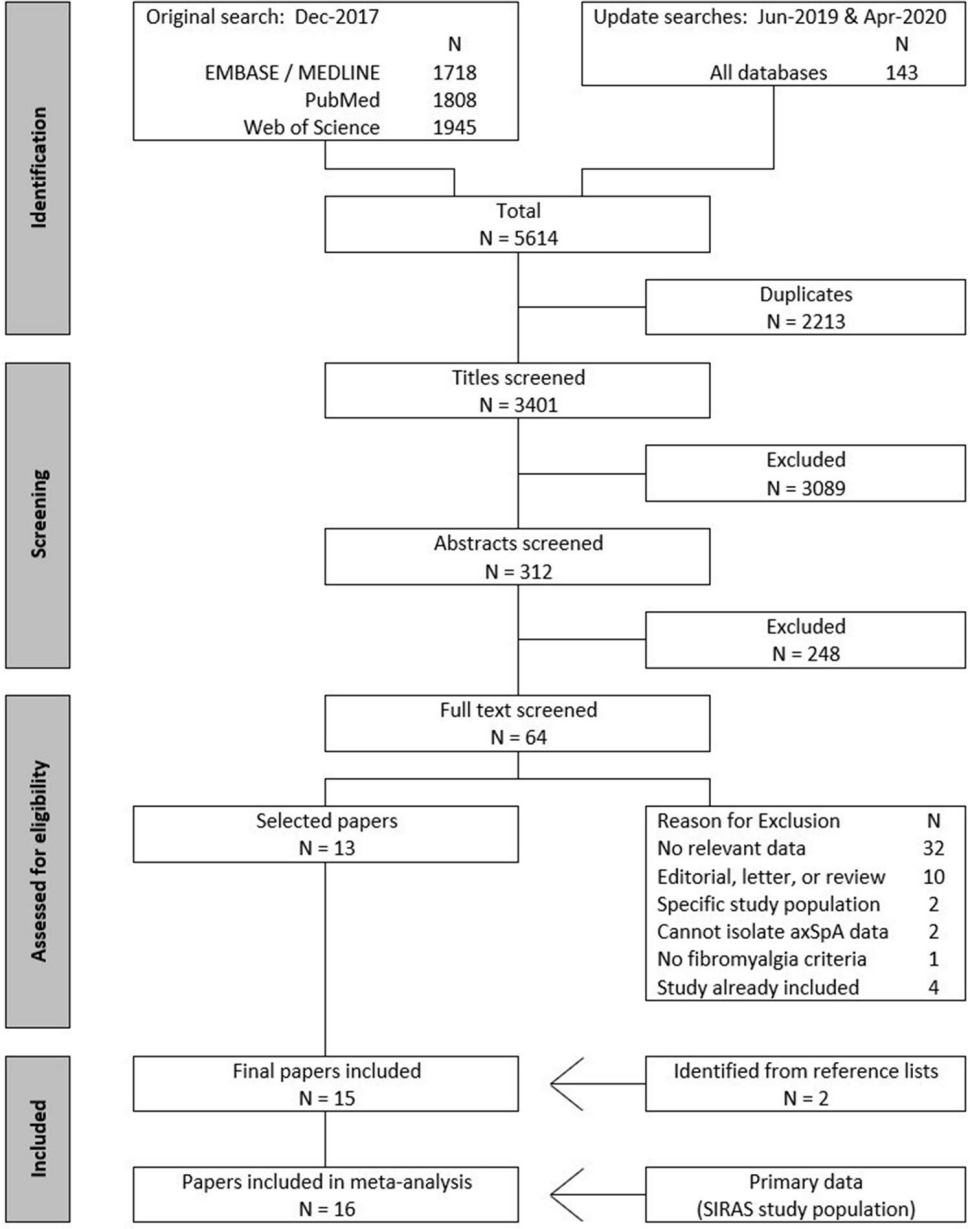
PRISMA flow diagram

From 3401 unique publications whose titles were screened, 312 proceeded to abstract screening, a process which identified 64 full text articles to screen for eligibility (Fig. 1). Of these, 13 manuscripts were deemed eligible for inclusion in the review. Two further manuscripts were added after screening the reference lists of other papers to provide a total of 15 eligible manuscripts/studies.

Eligible studies included 4725 patients, mainly from Europe or Israel. Generally, inclusion criteria for older studies were defined using clinical diagnosis, or modified New York classification of AS; whereas more recent papers used ASAS criteria for axSpA either alone or as well as AS. Although most studies were published after the 2010 revision of the ACR fibromyalgia classification criteria, most studies measured the outcome using the ACR-1990 criteria. A summary of the characteristics of the included studies is shown in Table 1.

**Table 1:** Key characteristics of included studies

Study	Sampling frame	Sample size, country	Phenotype		Prevalence of fibromyalgia (95% CI)^a^
			axSpA	Fibromyalgia Fibromyalgia	
Studies identified by systematic review					
Aloush et al. [21]	Consecutive patients from single centre	36, Israel	mNY criteria for AS	ACR-1990 criteria	25.0% (12.1–42.2%)
Almódovar et al. [11]	Random sample from 10 centres participating in national registry	462, Spain	mNY criteria for AS	ACR-1990 criteria	4.1% (2.5–6.3%)
Azevedo et al. [22]	Patients from single centre	71, Brazil	mNY criteria for AS	ACR-1990 criteria	15.5% (8.0–26.0%)
Demirdal et al. [23]	Patients from single centre	77, Turkey	mNY criteria for AS	ACR-1990 criteria	16.9% (9.3–27.1%)
Wallis et al. [19]	Consecutive patients from single centre	712, Canada	Clinical diagnosis, but ASAS criteria for axSpA were applied	Not stated	*mNY criteria for AS* 6.1% (4.4–8.2%) *ASAS criteria for nr-axSpA* 13.7% (10.9–30.1%)
Haliloglu et al. [24]	Consecutive patients from single centre	119, Turkey	Clinical diagnosis	ACR-1990 criteria	12.6% (7.2–19.9%)
Salaffi et al. [25]	Patients from single centre	211, Italy	mNY criteria for AS	ACR-2010 criteria	12.8% (8.6–18.1%)
Bello et al. [26]	Consecutive patients from single centre	182, France	Clinical diagnosis, but ASAS criteria for axSpA were applied	FiRST questionnaire (score ≥ 5/6)	*ASAS (imaging) criteria* 21.3% (15.1–28.8%) *ASAS (clinical) criteria* 18.8% (7.2–36.4%)
Wach et al. [27]	Consecutive patients from single centre	81, France	ASAS criteria for axSpA	ACR-1990 criteria	14.8% (7.9–24.4%)
Fan et al. [20]	Consecutive patients from single centre	201, France	mNY criteria for AS, or ASAS criteria for axSpA	a. ACR-1990 criteria b. Physician diagnosis	*mNY criteria for AS* a. 6.6% (3.0–12.1%) b. 7.3% (3.6–13.0%) *ASAS criteria for axSpA* a. 23.4% (13.8–35.7%) b. 37.5% (25.7–50.5%) *All participants* a. 11.9% (7.8–17.2%) b. 16.9% (12.0–22.8%)
Macfarlane et al. [2]	Patients participating in national registry	1504, UK	mNY criteria for AS, or ASAS criteria for axSpA	ACR-2011 criteria	*mNY criteria for AS* 19.7% (17.6–21.9%) *ASAS (imaging) criteria* 20.9% (19.0–22.9%) *ASAS (clinical) criteria*^b^ 9.2% (3.5–19%) *All participants* 20.7% (18.7–22.8%)
Monti et al. [28]	Consecutive patients, stable on TNFi, from two centres	218, Italy	Radiographic arm of the ASAS criteria for axSpA, including definitions for nr-axSpA^c^	Clinical diagnosis	*mNY criteria for AS* 16.4% (11.1–22.9%) *ASAS (imaging) criteria* 19.7% (14.7–25.6%)
Baraliakos et al. [18]	Patients from multiple centres	200, Germany	ASAS criteria for axSpA (including imaging as per the mNY criteria for AS)	a. ACR-1990 criteria b. ACR-2010 criteria	*mNY criteria for AS* a. 29.0% (20.4–38.9%) b. 19.0% (11.8–28.1%) *ASAS (imaging) criteria* a. 23.2% (13.9–34.9%) b. 7.2% (2.4–16.1%) *ASAS (clinical) criteria* a. 9.7% (2.0–25.8%) b. 9.7% (2.0–25.8%) *ASAS criteria for axSpA (nr-axSpA)* A 19.0% (11.8–28.1%) B 8.0% (3.5–15.2%) *All participants* a. 24.0% (18.3–30.5%) b. 13.5% (9.1–19%)
Moltó et al. [13]	Patients on TNFi, from multiple centres	526, France and Algeria	Clinical diagnosis	a. ACR-1990 criteria b. FiRST questionnaire (score ≥ 5/6)	*ASAS (imaging) criteria* a. 13.4% (10.2–17.2%) b. 38.2% (33.4–43.2%) *ASAS (clinical) criteria* a. 10.9% (3.6–23.6%) b. 34.8% (21.4–50.2%) *ASAS criteria for axSpA* a. 13.2% (10.1–16.7%) b. 37.9% (33.3–42.6%) *Clinical diagnosis, not meeting ASAS criteria* a. 32.9% (23.1–44.0%) b. 41.2% (30.6–52.4%) *All participants* a. 16.3% (13.3–19.8%) b. 38.4% (34.2–42.7%)
Rençber et al. [31]	Patients from single centre	125, Turkey	ASAS criteria for axSpA	ACR-2010 criteria	29.6% (21.8–38.4%)
Primary data					
SIRAS study population [7]	National registry	489, Scotland	Clinical diagnosis	ACR-2011 criteria	26.6% (22.7–30.7%)

*ACR* American College of Rheumatology, *AS* Ankylosing spondylitis, *ASAS* Assessment for Spondyloarthritis International Society, *axSpA* Axial Spondyloarthritis, *FiRST* Fibromyalgia Rapid Screening Tool, *mNY* Modified New York, *nr-axSpA* Non-radiographic Axial Spondyloarthritis, *TNFi* TNFα inhibition

^a^Exact confidence intervals computed using the method described in: Clopper and Pearson (9). Operationalised as per: http://sigmazone.com/binomial-confidence-intervals

^b^Manuscript gives prevalence 9.5%. 9.2% is a corrected estimate

^c^Manuscript states that “All patients fulfilled the radiographic arm of the ASAS criteria for axSpA, including definitions for nr-axSpA”. However, there is no ‘radiographic’ arm as such – there are ‘imaging’ and ‘clinical’ arms. Thus, we assume that where authors refer:

i. To AS, they are referring to patients with positive x-ray imaging (as per the mNY criteria for AS, which is incorporated within the ASAS imaging classification); and

ii. To nr-axSpA, they are referring to patients with positive MR imaging (as per the ASAS imaging classification) but not a positive x-ray

Prevalence of fibromyalgia in axSpA populations varied considerably between studies, and across different classification criteria both for axSpA and fibromyalgia. There was a ten-fold variation in prevalence, from 4.1% (95% CI 2.5–6.3%) using the ACR-1990 fibromyalgia classification criteria, among 462 patients from Spain with AS (modified New York criteria) [11], to 41.2% (30.6–52.4%) using the Fibromyalgia Rapid Screening Tool (FiRST) [12], among 526 French / Algerian patients with a clinical diagnosis of AS but who failed to meet any ASAS classification criteria [13].

### Scotland Registry for Ankylosing Spondylitis (SIRAS)

Data were available on 489 participants who completed the relevant follow-up questionnaire to allow determination of fibromyalgia. 71% of participants were male, and they had a mean age of 58 years (SD = 11), with mean (SD) disease activity and function scores of 4.1 (2.6) and 4.7 (3.1), respectively, as determined by the Bath Ankylosing Spondylitis Indices for Disease Activity (BASDAI) [14] and Function (BASFI) [15]. 145 (84%) of persons tested were HLA-B27 positive, and 130 participants met ACR-2011 research criteria for fibromyalgia (26.6%; 22.7–30.7%).

### Meta-analysis

**Fig. 2:**
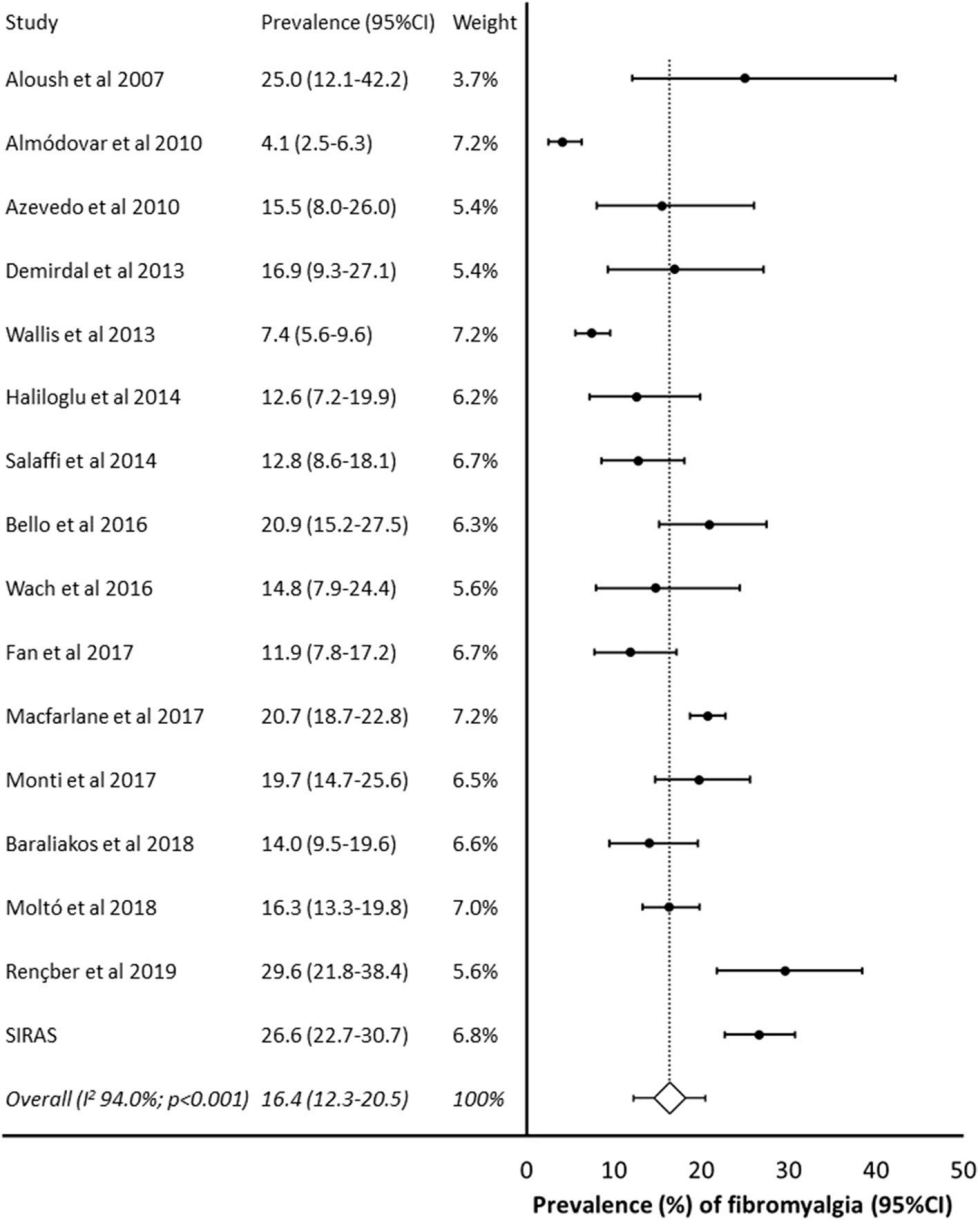
Prevalence of fibromyalgia (all studies)

**Fig. 3:**
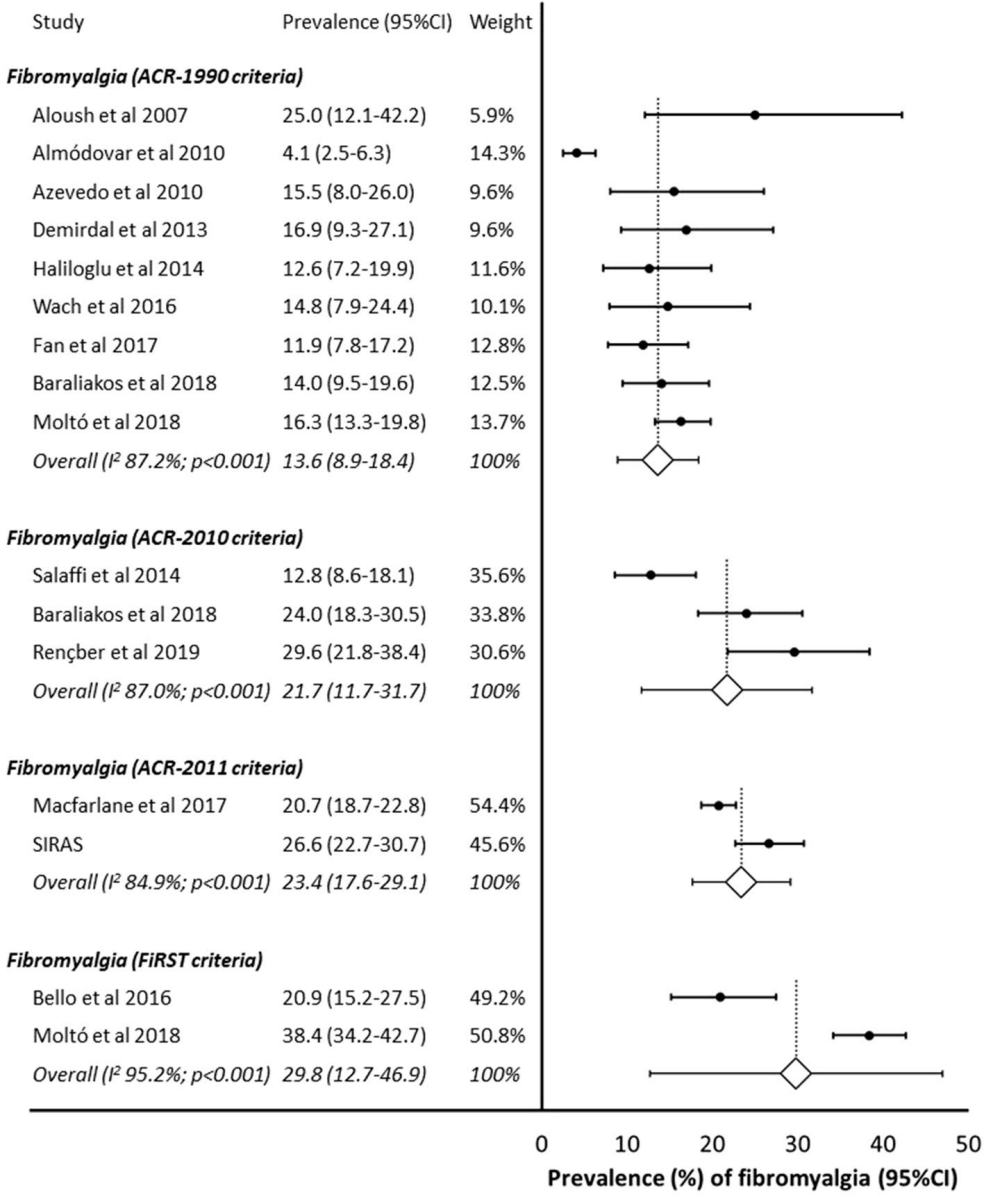
Prevalence of fibromyalgia (stratified by fibromyalgia classification criteria)

The combination of data from the systematic review, plus the SIRAS study population, allowed 5214 individuals, from 16 studies, to be included in the meta-analysis. There was evidence of considerable heterogeneity between studies (*I*^2^ = 94.0%; *p* < 0.001) so a random effects analysis was performed. Across all studies, the pooled prevalence of fibromyalgia was 16.4% (12.3–20.5%) (Fig. 2). Nine of these 16 studies defined fibromyalgia using the ACR-1990 criteria. Restricting the analysis to these nine (*N* = 1773) revealed a pooled prevalence of 13.6% (8.9–18.4%) (Fig. 3). Three studies (*N* = 536) were identified that used the ACR-2010 fibromyalgia classification criteria, giving a pooled estimate of 21.7% (11.7–31.7%), and two studies that used each of the ACR-2011 (*N* = 1993): 23.4% (17.6–29.1%); and FiRST criteria (*N* = 708): 29.8% (12.7–46.9%) (Fig. 3). A further three studies (*N* = 1131) provided data with fibromyalgia classified as per clinical diagnosis. The pooled prevalence was 14.4% (5.8–22.9%).

**Fig. 4:**
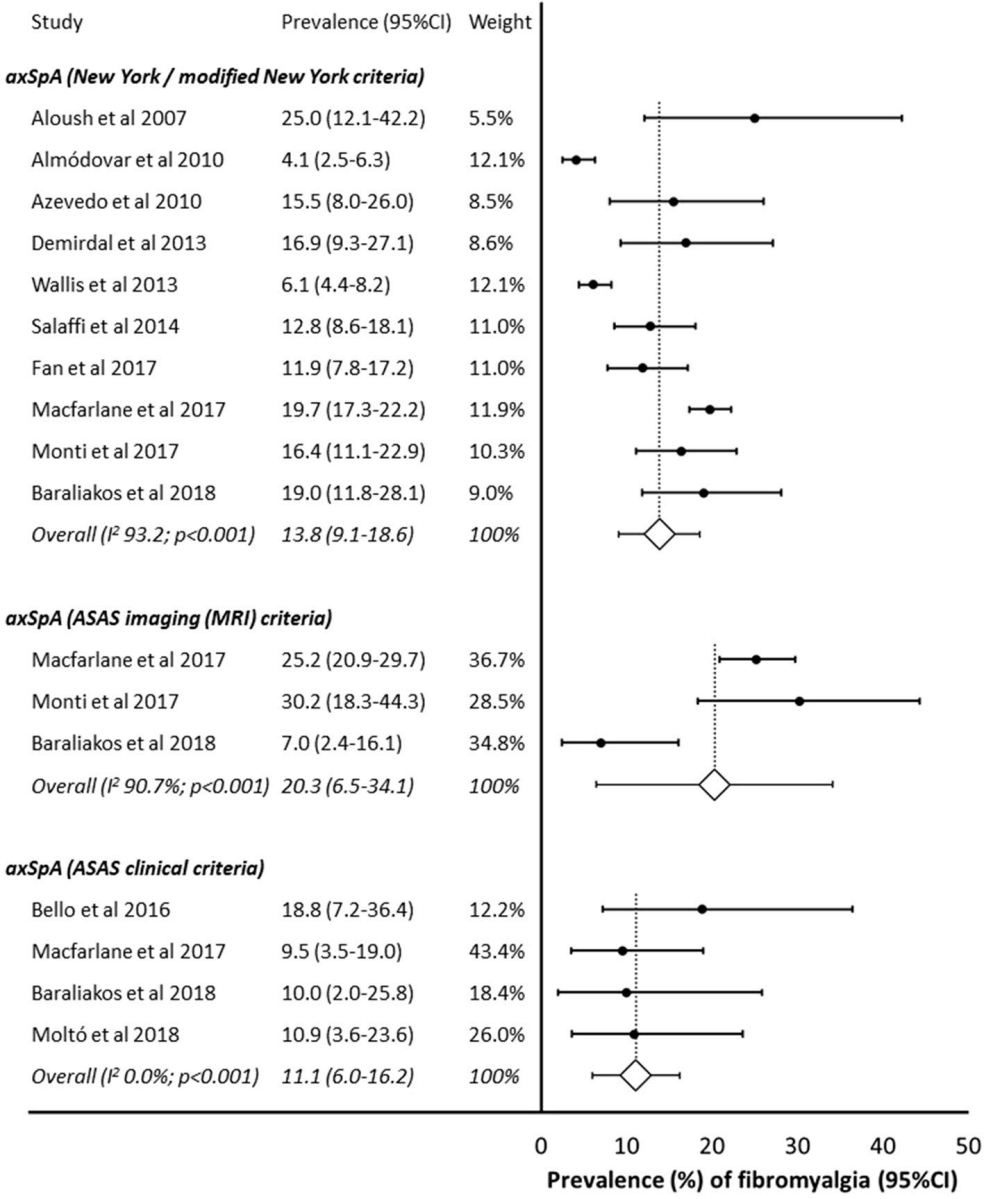
Prevalence of fibromyalgia (stratified by axSpA classification criteria)

Ten studies (*N* = 3003) presented data among patients with AS (New York or modified New York classification criteria), among whom the pooled estimate of fibromyalgia prevalence was 13.8% (9.1–18.6%) (Fig. 4). Prevalence was higher among the 520 patients from three studies who met ASAS imaging criteria for nr-axSpA: 20.3% (6.5–34.1%), but lower among those who only met the ASAS clinical criteria: 11.1% (6.0–16.2%). For the latter model, heterogeneity was low (*I*^2^ = 0%), so a fixed effects model was used for the combined estimate; 4 studies (*N* = 174).

## Discussion

Fibromyalgia in axSpA is common. This meta-analysis, including some hitherto unpublished results, shows clear and considerable variation in prevalence estimates when employing different fibromyalgia classification criteria (range 14–30%), and with different axSpA classification criteria (range 11–20%). However, overall, we have demonstrated that around one in six patients with axSpA also meet criteria for fibromyalgia.

Our review strategy was comprehensive, covered four main bibliographic databases and, although the initial search was conducted in December 2017, it was updated to April 2020. Other databases are available, such as Scopus, Web of Science and Google Scholar. While these were not included in the search, there is enormous overlap between different databases and the probability of missing a full-text peer-reviewed article relevant to the current review is low. In support of this, it is reassuring that only two additional studies were found from checking the reference lists of the included papers—and these were included in the review. Screening of manuscript titles was deliberately conservative (i.e. a manuscript was only excluded if the reviewer was confident that it did not contain relevant information) and the search allowed inclusion of studies of any language. For all non-English studies, an English language title and/or abstract was available which permitted screening based on content rather than language. No full text studies in a non-English language were eligible, so translation was not required. Various data were extracted from included studies. Where confidence intervals were presented in the original studies, they most commonly utilised the normal approximation of the binomial confidence interval. While this is acceptable for almost all purposes, in order that the data in Table 1 match the output from the meta-analysis, these were recomputed as ‘exact’ confidence intervals. Some papers only provided a prevalence estimate and total sample size. Thus, the number of participants with fibromyalgia was computed by applying the prevalence proportion to the total reported sample and rounding to no decimal places (because *N* must be a positive integer). This may have introduced a small degree of error where missing data was not reported, and this would have decreased the standard error of the prevalence estimate and increased the weight of the study in the meta-analysis. However, for the main findings this only applied to three studies, and a sensitivity analysis excluding these studies had little effect on the results (fibromyalgia prevalence 17.8% (12.7–22.9%)). This suggests that our approach has not introduced any major bias.

Over the last decade, the rheumatology community has moved away from defining fibromyalgia using widespread pain and widespread tenderness on palpation, as per the ACR-1990 criteria. Instead, more recent criteria have classified fibromyalgia based on the presence of widespread pain, fatigue, tiredness, cognitive problems, and other common symptoms. In total, five methods of classifying fibromyalgia were employed across the included studies: ACR-1990, 2010 and 2011, the FiRST, and clinical diagnosis—and often more than one per paper. However, in order to maintain independence of observations in the meta-analysis, only one estimate could be used in any single pooled estimate. Unsurprisingly, the most long-standing classification criteria, the ACR-1990 criteria, were employed most commonly, in nine out of 15 studies (*N* = 1773). Therefore, for the overall pooled estimate, data on ACR-1990 fibromyalgia took precedence. However, recent studies employing the ACR-2011 criteria in the UK have been large and, despite comprising only two studies, the total patient population (*N* = 1993) was larger. We have shown previously, in the general population, that the prevalence of fibromyalgia varies three-fold depending on whether the ACR-2011 or ACR-1990 criteria are employed (5.4% versus 1.7%) [29]. In the current study, the difference was less than two-fold, although the absolute magnitude of the difference was considerably greater (23% versus 14%).

More recent papers employed more recent fibromyalgia classification criteria and this, of course, is not unexpected. However, the stratification of analysis by fibromyalgia criteria was not a pre-specified analysis. Post hoc analyses are a potential concern in meta-analyses reporting treatment effects, and when stratification involves splitting *all* available participant data into sub-groups, by demographic characteristics, presence of comorbidities, etc. In the current review, sub-grouping was based simply on whether different classification criteria were available. It is important to note that fibromyalgia classification criteria do not constitute a clinical diagnosis, and many patients who meet classification criteria may not have been clinically diagnosed. However, previously work has shown that, despite this, while patients who fulfil the ACR-2011 criteria may not have elevated C-reactive protein levels compared to other patients, they do report more severe disease (higher disease activity and poorer function) as well as high fatigue, and poorer mental health and quality of life [2]. They do, therefore, represent a specific axSpA sub-group in whom additional management is warranted. We believe that the somatic symptoms component of the ACR-2011 fibromyalgia criteria may best distinguish those with/without fibromyalgia and indeed, there is evidence that symptoms severity score is a predictor of non-response to TNF inhibition [30].

One paper, by Moltó et al. [13], only included patients with axSpA in whom their treating physician had decided either to commence or switch TNF inhibition [13]. This clearly comprises a subset of all axSpA patients and is distinct from other included studies, which were largely patient registries, or series of consecutive patients. However, a sensitivity analysis, excluding the study by Moltó et al. [13] made little difference to the main findings (fibromyalgia prevalence 16.4%; 12.0–20.8%), again suggesting that no major bias was introduced through the inclusion of this study.

The prevalence of fibromyalgia varies markedly in persons with nr-axSpA with/without MRI evidence of sacroiliitis; the ASAS imaging versus clinical criteria. The current study does not address the US Food and Drug Administration’s concerns that patients with fibromyalgia may be inappropriately classified as nr-axSpA in the current study. We show that around 11% of patients who meet the ASAS clinical criteria for axSpA experience comorbid fibromyalgia, but we cannot comment on the proportion of patients with fibromyalgia who have axSpA. However, others have demonstrated this to be very low: Baraliakos et al. [18] found that, among 100 patients with fibromyalgia, only 2% fulfilled the ASAS criteria. Even among those who were HLA-B27 positive, prevalence was only 5%. It is also useful to remember that patients fulfilling the ASAS clinical criteria are those without a positive image for sacroiliitis. They therefore comprise not only those who are imaging negative, but also those in whom no image has been taken. Very few papers make this distinction.

Our findings are similar to those of Duffield and Miller et al. [4], who reported a pooled prevalence in AS of 13% (7–19%). These authors acknowledge the presence of additional papers combining radiological non-radiological criteria, although they did not attempt to combine these estimates. Here, we adopted a different approach. Recent thinking suggests that axSpA may manifest as a spectrum of inflammatory spinal disease and thus AS (radiographic axSpA) and nr-axSpA, with or without MRI findings, are conceptually part of the same disease entity. Although we also present separate prevalence estimates for the different sub-groups, we therefore believe that a single pooled estimate is entirely legitimate, and with additional studies plus the previously unpublished SIRAS data, we present the pooled prevalence estimate with the highest available precision.

However, there is still considerable uncertainty around some sub-group estimates. The pooled data for patients with MRI positive nr-axSpA is based on only 520 patients from three studies. As more data becomes available, the precision from this estimate will improve. Only two studies present data on three axSpA classification criteria in the same study population [2, 18] but there is no overlap in outcome measurement (fibromyalgia criteria). From the description of several studies presented here it’s clear that other data is available—some authors [19, 20] described their cohorts in the context of imaging and clinical arms separately—but do not currently provide separate estimates for fibromyalgia, nor data from which this can be computed.

In summary, fibromyalgia is a common comorbidity in axSpA, experienced by more than one in every six patients. Prevalence is similar between those with radiographic disease (14%) and those with various clinical features but who have no imaging evidence of sacroiliitis (11%), whereas prevalence is higher among those with MRI-positive nr-axSpA (20%). However, in all sub-classifications of axSpA fibromyalgia represents an important burden. Thus, for a sizeable proportion of the patient population, a focus solely on reducing the inflammatory aspects of disease are unlikely to yield optimal improvements in patient quality of life. Here we provide the rheumatologist with the current best evidence in terms of likely burden of fibromyalgia in his / her axSpA patient population which should be used to help direct resources in terms of patient management.
